# Identification and Characterization of *Eimeria tenella* Apical Membrane Antigen-1 (AMA1)

**DOI:** 10.1371/journal.pone.0041115

**Published:** 2012-07-19

**Authors:** Lianlian Jiang, Jiaojiao Lin, Hongyu Han, Hui Dong, Qiping Zhao, Shunhai Zhu, Bing Huang

**Affiliations:** Key Laboratory for Animal Parasitology, Ministry of Agriculture, Shanghai Veterinary Research Institute, Chinese Academy of Agricultural Sciences, Minhang District, Shanghai, China; University of Melbourne, Australia

## Abstract

Apical membrane antigen-1 (AMA1) is a micronemal protein of apicomplexan parasites that appears to be essential during the invasion of host cells. In this study, a full-length cDNA of AMA1 was identified from *Eimeria tenella* (Et) using expressed sequence tag and the rapid amplification of cDNA ends technique. EtAMA1 had an open reading frame of 1608 bp encoding a protein of 535 amino acids. Quantitative real-time PCR analysis revealed that EtAMA1 was expressed at higher levels in sporozoites than in the other developmental stages (unsporulated oocysts, sporulated oocysts and second-generation merozoites). The ectodomain sequence was expressed as recombinant EtAMA1 (rEtAMA1) and rabbit polyclonal antibodies raised against the rEtAMA1 recognized a 58-kDa native parasite protein by Western Blotting and had a potent inhibitory effect on parasite invasion, decreasing it by approximately 70%. Immunofluorescence analysis and immunohistochemistry analysis showed EtAMA1 might play an important role in sporozoite invasion and development.

## Introduction

Avian coccidiosis is the major disease of poultry infected by parasitic *Eimeria* spp. and inflicts severe economic losses on the poultry industry [1]. At present, conventional disease control strategies rely on prophylactic medication and immunization with live vaccines [2], [3]. Owing to the emergence of drug-resistant parasites and the difficulties associated with the use live vaccines, novel approaches to control coccidiosis are urgently needed [4]–[6]. Recent efforts to clone genes from *Eimeria* spp. for potential recombinant vaccines are directed toward developing an alternative strategy for the parasite control [7]. Although several recombinant vaccine candidate proteins have been proposed, effective recombinant subunit vaccines have not yet been formulated [8]–[10]. To develop effective recombinant vaccines, it is crucial to characterize various components of the parasite and to understand the nature of the host–parasite interaction.

The invasion of host cells by *Eimeria* spp. is a complex, multi-step process that begins with the apical attachment of the parasite to the host cell. This is followed by rapid internalization to form an intracellular, parasitophorous vacuole (PV) in which the newly invaded parasite becomes enclosed, enabling its survival within the host [11]. During the invasion process, specialized secretory organelles known as micronemes, rhoptries and dense granules deliver cargo proteins in a coordinated fashion. The secreted proteins are thought to have a central role in invasion and the establishment of infection [12], [13].

Apical membrane antigen 1 (AMA1), which is secreted by micronemes, was first identified as a conserved antigenic protein in the malaria parasite *Plasmodium knowlesi* [14]. In addition to rhoptry neck proteins (RONs), AMA1 is involved in the formation of the moving junction complex, which is a circumferential zone that moves backward and eventually pinches the PV from the host cell membrane [15]–[19]. *Plasmodium falciparum* AMA1 (PfAMA1) has been demonstrated to induce protective immunity against the parasite challenge in animal models [20]. Recently, AMA1 has been identified as immunoprotective proteins from other apicomplexan parasites, such as *Babesia*, *Toxoplasma*, *Neospora* and *Theileria* [21]–[24].

Recent developments in genomic and proteomic research have led to further insights into AMA1 from *Eimeria* spp. Expressed sequence tags (ESTs) of *Eimeria tenella* were analyzed and some EST sequences showed homology with AMA1 [25], [26]. The AMA1 protein was also detected in sporoziotes by proteomic comparison of four *E. tenella* life-cycle stages [27]. In 2011, one sequence of *Eimeria maxima* AMA1 was reported as a potential vaccine candidate [28]. However, no information has been available regarding the full-length cDNA and further characterization of *E. tenella* AMA1 (EtAMA1). *E. tenella* is one of the most virulent of seven *Eimeria* species that infect chickens. It develops in the intestinal ceca, provoking hemorrhage and, in severe cases, anemia and death owing to blood loss and shock [29].

Here, we report the cloning, sequencing and characterization of EtAMA1 and provide novel insights into the parasite invasion and development resulting from a detailed study of the expression of EtAMA1.

## Materials and Methods

### Parasite Propagation and Purification

The Shanghai strain of *E. tenella* was isolated from a sample collected in a chicken farm in Shanghai, China in 1985 and has been maintained in our laboratory. Parasites were propagated as previously described [30], by passage through 2-week-old coccidia-free chickens. Unsporulated oocysts were obtained from the cecal contents of chickens at day 8 post-infection (p.i.). A portion of the unsporulated oocysts was purified and stored in liquid nitrogen, and another portion was incubated in 2.5% potassium dichromate solution to induce sporulation. After sporulation, the sporulated oocysts were collected and purified. Sporozoites were prepared from cleaned sporulated oocysts by *in vitro* excystation and were purified by chromatography over columns packed with nylon wool and DE-52 cellulose [31]. Second-generation merozoites were collected from ceca at 110 h p.i. from chickens that had been inoculated with 1×10^5^ sporulated oocysts per bird. Isolation was carried out as previously described [32]. Isolated sporozoites and merozoites were stored in liquid nitrogen until required.

### Isolation of RNA and Amplification of Full-length cDNA

A single EST homologous to the AMA1 (GenBank accession number: BG929589) was selected for full-length amplification [25]. Total RNA was extracted from sporozoites of *E. tenella* by using Trizol reagent (Invitrogen, USA), according to the manufacturer’s protocol. The resultant RNA quality was analyzed by 1% agarose gel electrophoresis and visualization with ethidium bromide (EtBr) staining. Total RNA concentration was quantified by UV spectrophotometry (Eppendorf, Germany). Rapid amplification of the cDNA ends (RACE) was carried out with the GeneRacer ™ kit (Invitrogen) to obtain the full-length 5′- and 3′-termini sequences. Approximately 2 mg of total RNA was used to synthesize the 5′- and 3′-RACE-Ready cDNA. Sequences were amplified by Touchdown PCR with either the EtA1 or EtA2 gene-specific primers (Table 1) and with the GeneRacer 3′- or 5′- primers. Nested PCRs were performed with nested gene-specific primers and 3′- or 5′- nested GeneRacer primers (Table 1). Amplification products were electrophoresed through 1% agarose gels and single bands were extracted, purified, cloned into the pGEM-T Easy vector (Promega, USA), and propagated in *Escherichia coli* TOP10 (Invitrogen). The clones were sequenced using an ABI 3730 sequencer (Applied Biosystems, USA). The sequences of 5′RACE and 3′RACE were compared against the original EST sequence by using DNAstar software (Promega) to determine overlap. The full-length cDNA was then spliced accordingly. The entire sequence of AMA1 was PCR-amplified from the sporozoite cDNA library using primers SA1 and SA2 to ensure the correct sequence had been obtained. The sequence analysis was carried out as previously described [33].

**Table 1:** Primer sequences used in this study.

Primer ID	Primer sequences
EtA1	5′-AAGTGCGAGTCTAAAGGGGGCGGTGT-3′
Nested-EtA1	5′-GTGTCTTCATTGGGCTGGCCGTCG-3′
EtA2	5′-GAAGGGAAGTAGTAGCCGGGACT-3′
Nested-EtA2	5′-GGGACTTGTTGCTGCTTCGTCTTGAGAT-3′
SA1	5′-TCTTCTTCAACTCTGGCAAAAGC-3′
SA2	5′-GAAGTGAAAAGGCGAAAACT-3′
qRT-PCR sense primer (AMA1)	5′-GCTGACTCTGCTGCTCATTTG-3′
qRT-PCR antisense primer (AMA1)	5′-GGAATATCAAGAGACCGCAACTG-3′
18Sr RNA sense	5′-TGTAGTGGAGTCTTGGTGATTC-3′
18Sr RNA antisense	5′-CCTGCTGCCTTCCTTAGATG-3′
A1	5′-CGGGATCCGGGGTGCAGCACAA-3′
A2	5′-CGGAATTCGCCCCCTTTAGA-3′

### EtAMA1 Transcript Expression in Four Life Stages of *E. tenella*

Total isolated RNA from four life stages of *E. tenella* (unsporulated oocysts, sporulated oocysts, sporozoites and second-generation merozoites) was treated with DNase I (Invitrogen) to remove all DNA contamination. The quality and quantity of total RNA were assessed as described above. cDNA was generated by SuperScript II reverse transcriptase (Invitrogen) using random primers. Quantitative real-time PCR (qRT-PCR) was performed on a Rotor-Gene 3000 (Corbett Robotics, USA) using the SYBR1 green I dye method. Negative (no template) controls were included in each PCR run. Five positive controls of known concentration were included in every run to confirm consistent amplification. Finally, quantitation of relative differences in expression was performed using Rotor-Gene version 6.0.38 software (Corbett Research, Australia). Expression of the gene encoding 18S rRNA was used as a control. The relative mRNA expression was determined as the ratio of EtAMA1 to 18S rRNA. Primers for EtAMA1 and the 18S rRNA were designed by the Beacon Designer program (Corbett Robotics) (Table 1). Each reaction was carried out in triplicate, and the experiment was performed three times.

**Figure 1:**
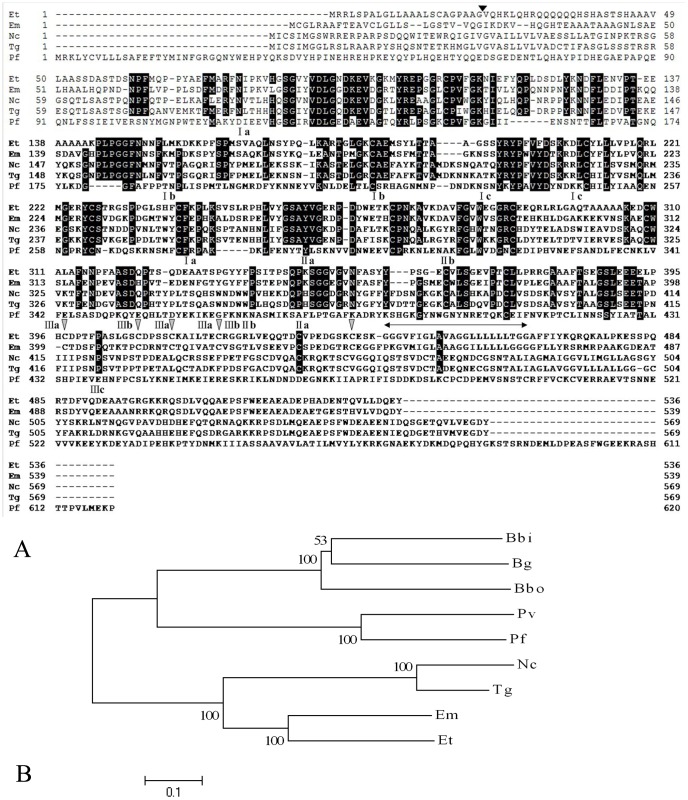
Characterization of the EtAMA1 gene. (A) Multiple-sequence alignment of AMA1 proteins from *E. tenella* (Et), *E. maxima* (Em), *N. caninum* (Nc), *T. gondii* (Tg) and *P. falciparum* (Pf). Strictly conserved residues are indicated with a black background, and five different cysteine residues with species of *Plasmodium* in DIII are indicated with a gray arrowhead. The cleavage site of the EtAMA1 putative signal peptide is indicated by an arrowhead, and the transmembrane region is indicated by ( ); cysteine residues that formed disulfide bonds in EtAMA1 are indicated by domain (I, II and III) and bond (a, b and c) designations according to TgAMA1. (B) Phylogenetic tree of AMA1 proteins from *E. maxima* (Em), *E. tenella* (Et), *N. caninum* (Nc), *T. gondii* (Tg), *P. falciparum* (Pf), *P. vivax* (Pv), *B. bovis* (Bbo), *B. gibsoni* (Bg),and *P. bigemina* (Pbi).

### Expression and Purification of Recombinant Proteins

The sequence-encoding ectodomain of EtAMA1 (amino acids 24 Val to 446 Glu) was amplified with A1 and A2 primers, which incorporated *BamH* I and *EcoR* I restriction sites (Table 1). The DNA product was cloned into the expression vector pGEX-6P-1 (Pharmacia Biotech, USA). The recombinant EtAMA1 (rEtAMA1) fused with a glutathione S-transferase (GST) tag was expressed in the *E. coli* BL21 strain, according to the manufacturer’s instructions. The denaturing and refolding of insoluble rEtAMA1 with urea and subsequent purification were performed as described previously [34].

**Figure 2:**
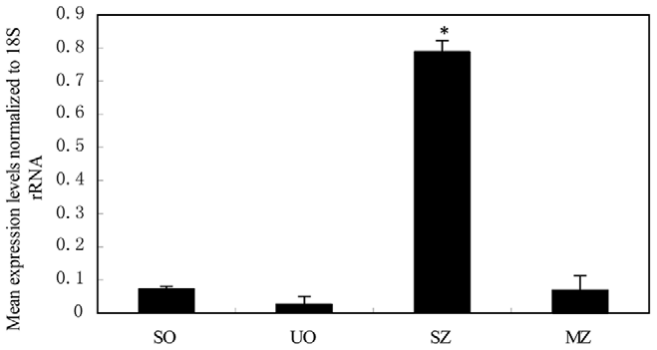
EtAMA1 mRNA expression levels in different developmental stages of *E. tenella* as revealed by SYBR Green quantitative real-time PCR. EtAMA1 mRNA levels in sporulated oocysts (SO), unsporulated oocysts (UO), sporozoites (SZ) and second-generation merozoites (MZ) were normalized to the 18S rRNA level of the corresponding stage. *P<0.05, n = 3.

### Production of Anti-rEtAMA1 Serum

Two 2-month-old rabbits were immunized by intraperitoneal (i.p.) injection with 0.2 mg of purified rEtAMA1 emulsified in Freund complete adjuvant (Sigma, USA). The rabbits were boosted three times at 2-week intervals with proteins emulsified in Freund incomplete adjuvant (Sigma). Eight days after the final immunization, serum was separated from the rabbits’ blood.

**Figure 3:**
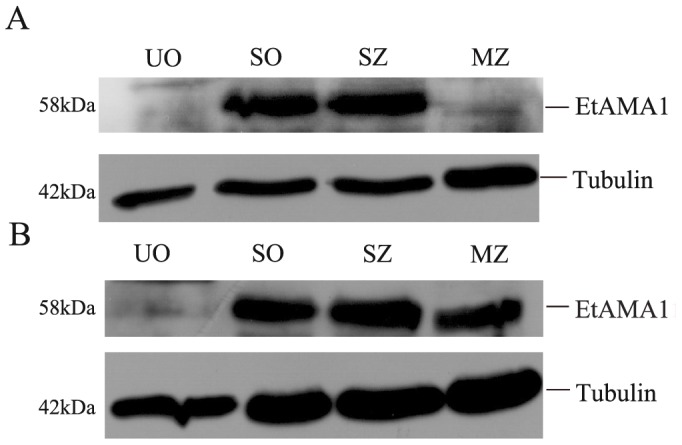
Western Blotting analysis of EtAMA1 in different life stages of *E. tenella* probed with anti-rEtAMA1 antibodies. (A) The cytosol proteins of parasites. (B) The membrane proteins of parasites. Lanes: UO, unsporulated oocysts; SO, sporulated oocysts; SZ, sporozoites; MZ, second-generation merozoites.

### EtAMA1 Expression Analysis in Parasites

To investigate the developmental expression of EtAMA1 in different stages, cytosol proteins and membrane proteins of unsporulated oocysts, sporulated oocysts, sporozoites and second-generation merozoites were, respectively, prepared by a Membrane and Cytosol Protein Extraction Kit (Beyotime, China). Proteins concentrations were determined with a BCA Protein Assay Kit (Beyotime). Then parasite protein extracts of each stage (150 µg) were subsequently separated on 12% SDS-PAGE. Western Blotting assays were performed according to standard procedures [29]. Anti-rEtAMA1 antibodies were used 1∶100 diluted and mouse monoclonal anti-tubulin antibodies (Beyotime) diluted 1∶2000 were used as controls. Secondary antibodies conjugated to horseradish peroxidase conjugated (HRP) (Sigma) were added 1∶5000 diluted.

**Figure 4:**
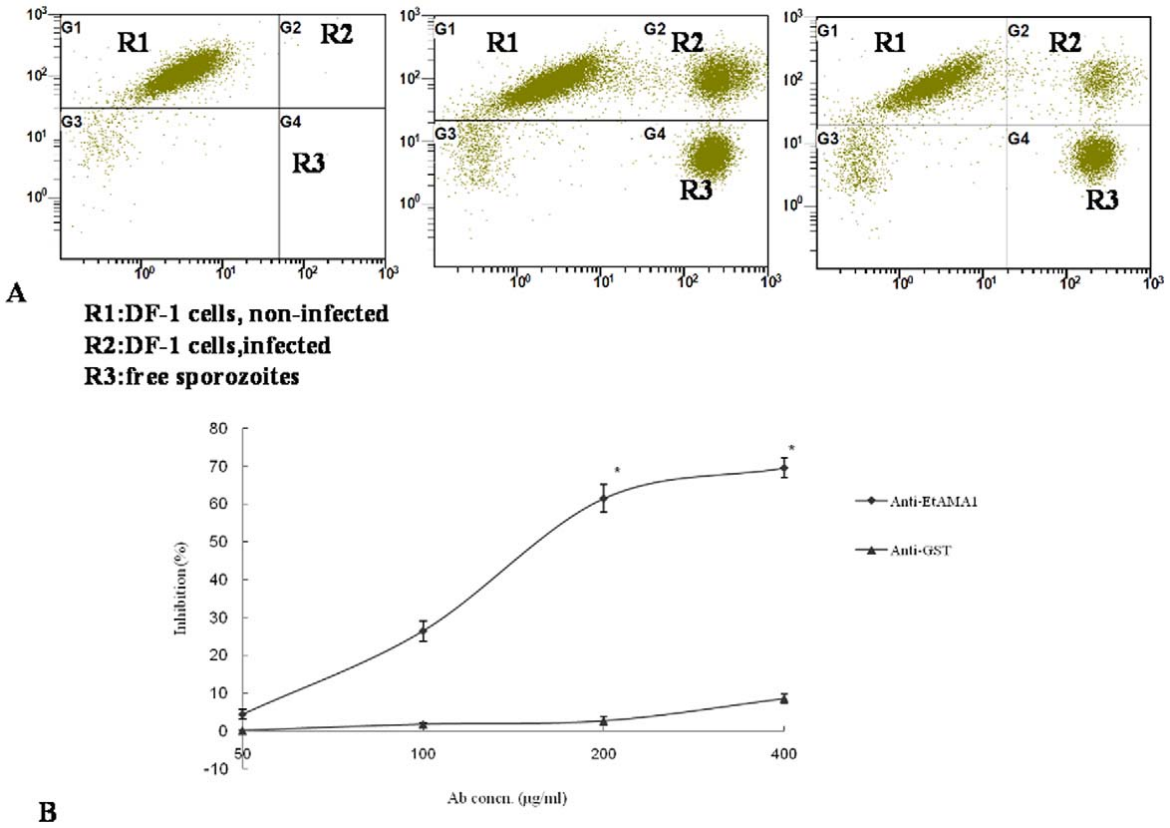
Inhibition of sporozoite invasion *in vitro* by antibodies specific to rEtAMA1. (A) Representative flow cytometry charts used to determine the inhibitory activity of the antibody on labeled sporozoites of *E. tenella*. Left panel, non-infected cells; Middle panel, infected cells as a negative control; right panel: infection with sporozoites preincubated with inhibitory antibodies of rEtAMA1 at a concentration of 400 µg/mL. (B) Dose-dependent inhibition of sporozoite invasion by antibodies specific to rEtAMA1 and GST. The results are representative of three individual experiments, and the error bars indicate standard deviations. (*) According to the Student’s t-test, the differences between the treatment with anti-GST antibodies and anti-rEtAMA1 antibodies at the same IgG concentration were significant (P<0.05).

### Inhibition of Host-cell Invasion *in Vitro*

The chicken embryo fibroblast cell line DF-1 (ATCC) was used for the inhibition assays [35]. The antibodies were purified with Protein A Agarose (Beyotime). Sporozoites were labeled using the dye carboxyfluorescein diacetate, succinimidyl ester (CFDA SE, Beyotime) according to the manufacturer’s instructions. The labeled sporozoites (1×10^5^) were resuspended in 1 mL of Dulbecco’s modified Eagle’s medium (DMEM; Gibco, USA) containing 50, 100, 200 and 400 µg/mL of anti-rEtAMA1 antibodies, and incubated 2 h at 37°C. Anti-GST IgG antibodies and parallel wells without the antibodies were used as controls. The sporozoites were then washed twice in sterile phosphate buffered saline (PBS) by centrifugation at 2000×g for 5 min and infected 1×10^5^ DF-1 cells in a 24-well plate (Corning, USA). After 12 h of culture at 41°C, cells were collected and analyzed by flow cytometry (Beckman Coulter, USA). The infected cells, non-infected cells and free sporozoites were gated using the software RXP for subsequent delineation and counting of the infected (containing the labeled sporozoites) and non-infected (fluorescence-free) cells. All assays were performed in triplicate. The deduced percentages of infected cells in the presence or absence of inhibitory antibodies were used for the calculation of the inhibition rates, as previously described [36].

### Immunofluorescence Analysis of EtAMA1 during the First Schizogony

Freshly excysted sporozoites were incubated for 1 h at 41°C in either PBS or complete DMEM and air-dried on a glass slide before fixation. Infected DF-1 cells were maintained on glass slides in a 6-well plate (Corning) and collected at different time points for fixation. Sporozoites or cells were fixed and permeabilized with 2% paraformaldehyde and 1% Triton X-100 for 10 min at room temperature and washed three times. Antibodies against rEtAMA1 were incubated with the purified recombinant protein GST at 37°C for 1 h to remove anti-GST antibodies and then incubated (1∶100 dilution) with the cells or sporozoites for 2 h at 37°C. After being washed three times, goat anti-rabbit IgG (H+L) fluorescein isothiocyanate (FITC)-conjugated antibody (1∶1000 dilution, Sigma) was added to the glass slides. Nuclei were labeled with 10 µg/mL of 4′,6-diamidino-2-phenylindole (DAPI) (Beyotime) for 5 min and washed five times. All dilutions and washes were performed in 0.05% Tween-20 PBS. Before observations under ﬂuorescence microscopy (Nikon, Japan), 10 µL of 1,4-diazabicyclo[2.2.2]octane (DABCO; Sigma) were added.

**Figure 5:**
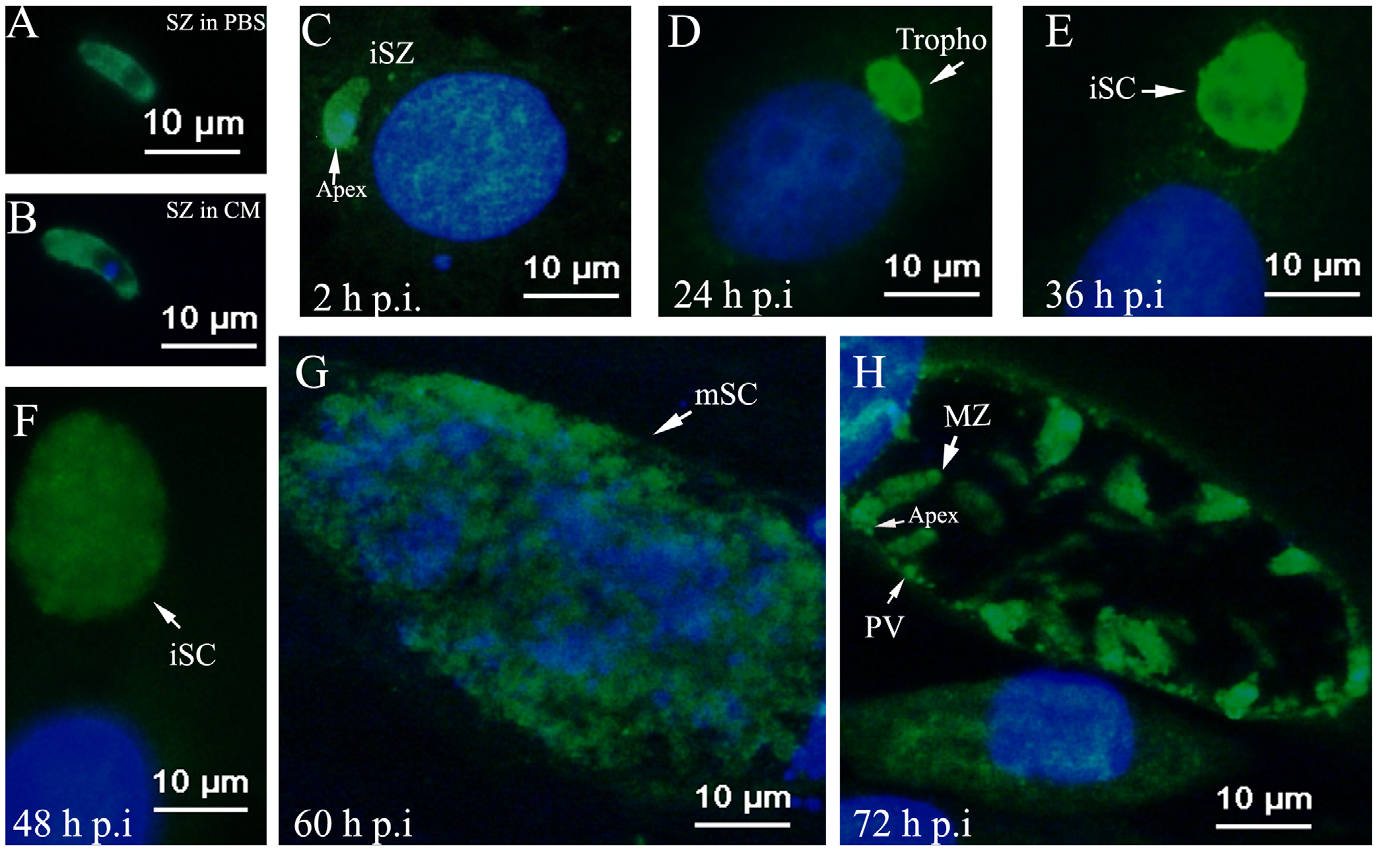
EtAMA1 localization in DF-1 cell infection as visualized by immunofluorescence analysis. Details of parasites immuno-stained with anti-rEtAMA1 antibodies, visualized with FITC (green) and counter-stained with DAPI (blue). (A) Sporozoites (SZ) were incubated in PBS or (B) complete medium (CM) at 41°C. Infected DF-1 cells were collected at different time points p.i. (C) 2-h p.i., intracellular sporozoites (iSZ); (D) 24-h p.i., intracellular trophozoite (Tropho); (E) 36-h p. i., immature schizont (iSc); (F) 48-h p.i., immature schizont (iSc); (G) 60-h p.i., mature schizont (mSc); (H) 72-h p.i., merozoites (MZ).

### Immunohistochemistry Analysis of EtAMA1 during the Parasitic Life-cycle

Three-day-old chickens were infected with 1×10^4^ sporulated oocysts. Their ceca were collected at 24-h intervals and fixed with 4% paraformaldehyde in PBS at room temperature. As controls, the ceca of uninfected chickens were also collected at the same time points. The ceca were embedded within optimal cutting temperature (OCT) compound mounted in a cryostat for production of a rapid (frozen) section 10-µm thick for immunohistochemistry analysis. Second-generation merozoites purified from the ceca of chickens were also prepared for observation. The samples were dyed using the immunofluorescence analysis procedure described above.

## Results

### Cloning and Molecular Characterization of EtAMA1

The full-length cDNA of EtAMA1 was 2349 bp (GenBank accession number:**JN032081**), including a single open reading frame (ORF) of 1608 bp, encoding a polypeptide of 535 amino acid residues. SignalP program analysis revealed that the N-terminus of EtAMA1 contained a signal peptide of 23 amino acids, with a hypothetical cleavage site located between glycine and valine. The EtAMA1 composed an ectodomain (from 24 to 446 aa), a transmembrane domain (from 447 to 469 aa) and a cytoplasmic domain (from 470 to 535 aa), as predicted using TMHMM2.0 (Figure 1A).

**Figure 6:**
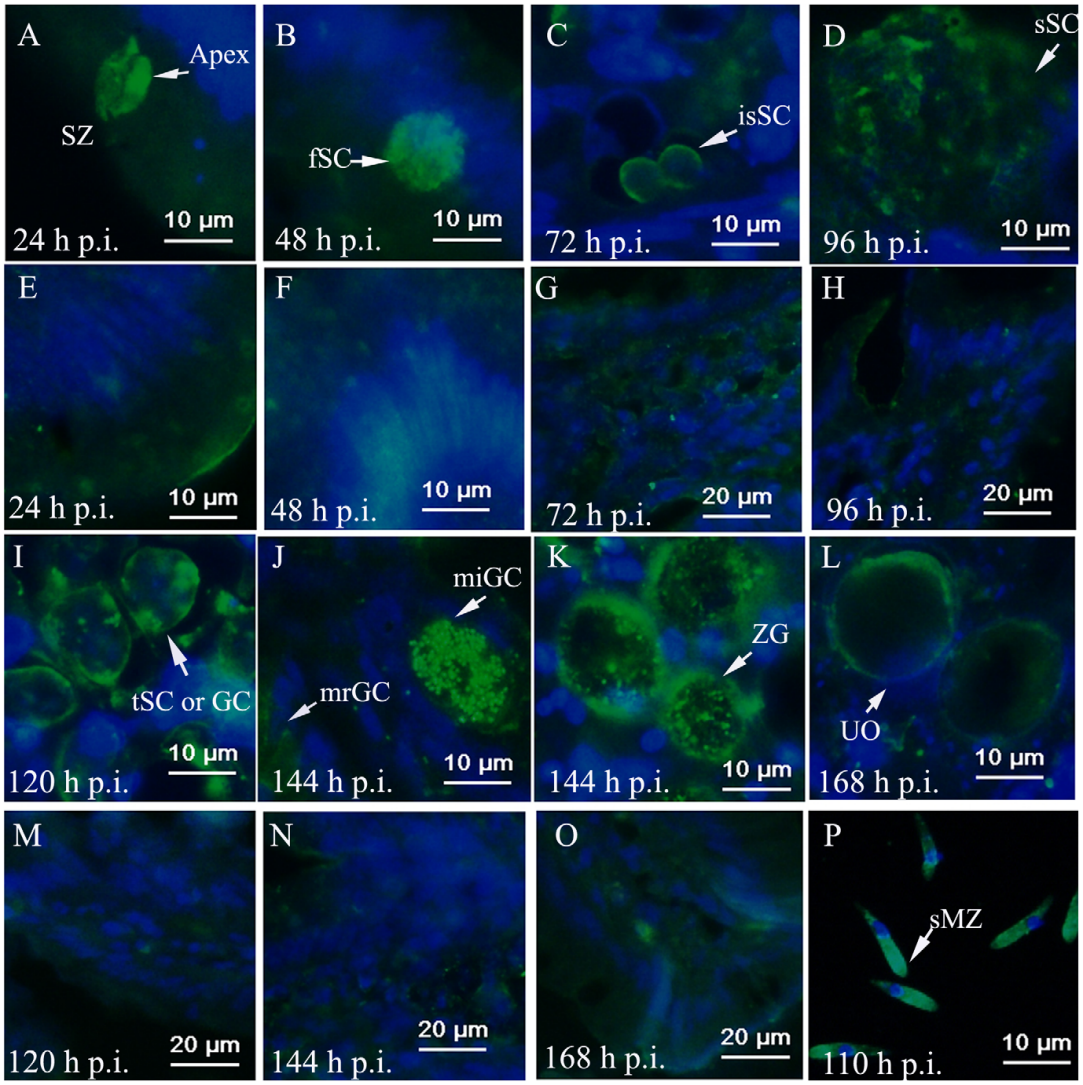
EtAMA1 localization in infected ceca as visualized by immunohistochemistry analysis. Details of parasites immuno-stained with anti-rEtAMA1 antibodies, visualized with FITC (green) and counter-stained with DAPI (blue), while the normal ceca collected at the same time point as controls. (A) 24-h p.i., sporozoites (SZ); (B) 48-h p.i., first-generation schizonts (fSC); (C) 72-h p.i., immature second-generation schizonts (isSC); (D) 96-h p.i., second-generation schizonts (sSC); (E) 24-h p.i., normal ceca; (F) 48-h p.i., normal ceca; (G) 72-h p.i., normal ceca; (H) 96-h p.i., normal ceca; (I) 120-h p.i., third-generation schizonts (or gametocytes) (tSC or GC); (J) 144-h p.i., microgametocytes (miGC) and macrogametocytes (mrGC); (K) 144-h p.i., zygotes (ZG); (L) 168-h p.i., unsporulated oocysts (UO); (M) 120-h p.i., normal ceca; (N) 144-h p.i., normal ceca; (O) 168-h p.i., normal ceca; (P) 110-h p.i., second-generation merozoites purified from ceca (sMZ).

To determine the phylogenetically conserved functional regions of EtAMA1, its deduced amino acid sequence was aligned with the AMA1 amino acid sequences from eight apicomplexan parasites [*E. maxima* (CBL80633.1), *Toxoplasma gondii* (XP_002364854), *Neospora caninum* (CBZ53079), *P. falciparum* (AAC15773.1), *P. vivax* (ACY68740), *B. bovis* (ACM44019), *B. bigemina* (BAH22706) and *B. gibsoni* (ABD04040)]. The phylogenetic tree was constructed by MEGA 4.0 software (Figure 1B). As calculated using Needleman-Wunsch global alignment, the highest amino acid sequence homology between EtAMA1 and EmAMA1 was 68.6% and the lowest homology between EtAMA1 and *B. bovis* AMA1 was 36.1%. Sixteen cysteine residues were conserved in *E. maxima* and *E. tenella*, whereas eight cysteine residues in the putative domains I and II were conserved in *E. tenella*, *E. maxima*, *T. gondii*, *N. caninum* and *P. falciparum,* as calculated using Genetyx software (Development Co., Ltd, Japan) (Figure 1A).

### EtAMA1 Transcript Expression in Four Life Stages of *E. tenella*

The expression of EtAMA1 showed high variation stages among the four life stages of *E. tenella*. EtAMA1 transcripts were abundant in sporozoites (30-fold higher than in unsporulated oocysts) (P<0.05) and only moderately expressed in sporulated oocysts and second-generation merozoites (no more than three-fold higher than in unsporulated oocysts) (Figure 2).

### EtAMA1 Protein Expression in Different Life Stages of *E. tenella*

The presence of EtAMA1 in unsporulated oocysts, sporulated oocysts, sporozoites and second-generation merozoites was determined by immunoblotting using antibodies obtained from rabbits immunized with rEtAMA1 as described above. Anti-tubulin monoclonal antibodies were used as controls to reveal a similar quantity of total proteins in all samples. Western Blotting of the cytosol proteins revealed that anti-rEtAMA1 antibodies strongly labeled the same 58-kDa bands in sporulated oocysts and sporozoites, but weaker reactivity in unsporulated oocysts and second-generation merozoites (Figure 3A). The membrance proteins probed with anti-rEtAMA1 antibodies showed EtAMA1 had high expression in sporulated oocysts, sporozoites and second-generation merozoites, while little expression in unsporulated oocysts.

### Anti-rEtAMA1 Antibodies Inhibited Host-cell Invasion of *E. tenella* Sporozoites

The polyclonal antibodies derived from rEtAMA1-vaccinated rabbits were tested for the ability to inhibit the invasion of cultured DF-1 cells with *E. tenella* sporozoites. Representative flow cytometry charts used to determine the inhibitory activity of the anti-rEtAMA1 antibodies on labeled sporozoites were presented in Figure 4A. There was an obvious distinction between cells infected with sporozoites pre-incubated without antibodies (as a negative control) and those infected with sporozoites with inhibitory anti-rEtAMA1 antibodies at a concentration of 200 µg/mL. Pretreatment with the anti-rEtAMA1 antibodies significantly decreased the invasion capacity of sporozoites, and the observed inhibition effect was dose dependent. Under the experimental conditions, the inhibition plateau of 69±2.55% was reached at an antibody concentration of 400 µg/mL. By comparative analysis, anti-GST antibodies did not have a significant effect on invasion (8.5±1.23%) (Figure 4B).

### EtAMA1 Localization during *in Vitro* Infection by Immunofluorescence Analysis

Anti-rEtAMA1 antibodies were applied to detect the localization of EtAMA1 in sporozoites and during the first schizogony. EtAMA1 exhibited a homogenous distribution pattern throughout the membrane of sporozoites incubated in PBS (Figure 5A). By contrast, when sporozoites were incubated in culture medium, EtAMA1 expression increased (Figure 5B). After sporozoites invaded host cells, the localization of EtAMA1 was mainly on the apical end of the parasites (Figure 5C). Observations 24 h p.i. showed that EtAMA1 protein expression increased and was distributed in trophozoites (Figure 5D, E). The labeled EtAMA1 eventually became uniformly dispersed in immature and mature schizonts (Figure 5F, G). After merozoites were released from host cells, EtAMA1 was translocated to the merozoite surface, although some remained in the PV membrane (Figure 5H).

### EtAMA1 Localization during *in Vivo* Infection by Immunohistochemistry Analysis

When sporozoites adhered to cecal epithelial cells, EtAMA1 was expressed on the apical end of the parasites (Figure 6A). As sporozoites developed into first-generation schizonts, EtAMA1 was expressed in first-generation merozoites (Figure 6B). After the parasites developed into immature second-generation schizonts, the expression of EtAMA1 was mainly around the schizont membrane (Figure 6C). When second-generation schizonts were mature, EtAMA1 was located in merozoites (Figure 6D). Once the parasites had developed into third-generation schizonts (or gametocytes), EtAMA1 expression was increased and mainly around the schizont/gametocyte membrane (Figure 6I). Interestingly, when the parasites entered into the stage of sexual reproduction, the expression of EtAMA1 was significantly increased in microgametocytes and decreased in macrogametocytes (Figure 6J). Simultaneously, EtAMA1 became concentrated in the cytoplasm of fertilized macrogametes as small defined regions and was also highly expressed around the membrane of the parasites in the contents of ceca (Figure 6K). However, as the parasites developed into unsporulated oocysts, EtAMA1 expression was reduced and was only detected around some oocysts wall (Figure 6L). In free second-generation merozoites, EtAMA1 was expressed mainly at the apical end of the parasites (Figure 6P).

## Discussion

In the present work, we described, for the first time, the molecular characterization of AMA1 in *E. tenella*. The full-length cDNA of EtAMA1 was 2349 bp, with a 1608 bp ORF encoding a protein of 535 amino acids. The 5′ untranslated region was located 469 bp upstream of the putative start codon (ATG). The 3′ untranslated region of 267 nucleotides ended at a poly (A) tail. These sequence characteristics all indicated that a full-length cDNA had been obtained. Phylogenetic comparison of the deduced amino acid sequence of the EtAMA1 gene with the amino acid sequences of AMA1 proteins from other apicomplexan parasites [*E. maxima* (EmAMA1), *T. gondii* (TgAMA1), *N. caninum* (NcAMA1) and *P. Falciparum*(PfAMA1)] demonstrated that each AMA1 sequence clustered depending on their genus (Figure 1B). The EtAMA1and EmAMA1 had higher amino acid sequence identities (68.6%) than with other apicomplexan AMA1 proteins. Moreover, The AMA1 proteins of *Eimeria* were closer to those of species of *Toxoplasma* and *Neospora* than of *Plasmodium* and *Babesia*. These results suggest that *Eimeria* spp. have a closer evolutionary relationship with *T. gondii* and *N. caninum* than with other apicomplexan species [37].

AMA1 proteins are type I integral transmembrane proteins that are highly conserved and present in all *Plasmodium* [14], *Toxoplasma* [21], *Babesia* [22] and *Neospora* [23] species. Analysis of the amino acid sequences revealed that full-length EtAMA1 contained a predicted transmembrane region (from 479 to 501 bp) near the C terminus, as seen in other AMA1 proteins. The predicted signal peptide of EtAMA1 contained 23 amino acids, which were associated with the secretory pathway. Many of the conserved and polymorphic residues in AMA1 proteins have been reported. EtAMA1 also contained 16 cysteine amino acid residues, which contribute to disulfide bonding, the pattern of which prompted the suggestion that the mature ectodomain folds as an N-terminal pro-sequence and three domains (DI, DII and DIII) [38]. Although there were many differences between the protein sequence of EtAMA1 and EmAMA1, both contained 16 highly conserved cysteines and exhibited identical localization. TgAMA1 and NcAMA1 included 13 cysteine residues with EtAMA1. The AMA1 proteins of *Plasmodium* spp. were more distantly related to those of *E. tenella*, *E. maxima*, *T. gondii* and *N. caninum*, but all the AMA1 proteins contained highly conserved eight cysteine amino acid residues in DI and DII which belong to the PAN module superfamily, which is commonly associated with carbohydrate or protein receptor-binding functions [38]. These results revealed that the number and localization of cysteine residues on the sequences of the AMA1 proteins might be conserved depending on their genus [23]; moreover, the differences in localization of the cysteine residues usually occurred in the DIII domain.

The correct folding of AMA1, as in case of *Plasmodium* antigens, has been shown to be crucial for its immunological activity [39]. In addition, the PfAMA1 ectodomain expressed in *E. coli* can be correctly folded and highly immunogenic [40]. In this study, the cDNA fragment of the EtAMA1 ectodomain was expressed in *E. coli*. The rEtAMA1 was initially insoluble; however, after refolding and purification, the rEtAMA1 became more soluble. The antibodies raised against sporozoites were reactive with rEtAMA1(date not show); Moreover, anti-rEtAMA1 antibodies inhibited host-cell invasion by the parasites *in vitro* and recognized the parasitic AMA1, as shown by Western Blotting, immunofluorescence analysis and immunohistochemistry analysis. These observations suggest that the refolded rEtAMA1 has immunological activity.

In our study, both qRT-PCR and Western Blotting demonstrated that the newly identified EtAMA1 was constitutively expressed in all four developmental stages. Proteomic comparison of *E. tenella* showed EtAMA1 was found in sporozoites and EtAMA2 was found in merozoites [27]. As Western Blotting analysis of second-generation merozoites showed EtAMA1 expression was little in soluble proteins, high in membrance proteins (Figure 3), soluble proteomic analysis of merozoites might miss the menbrance EtAMA1. There was one conflicting data for RNA versus protein levels of EtAMA1 expression in sporulated oocysts. As we know, oocysts of *Eimeria* spp. are able to persist in the environment for years by oxidation of lipids supporting the metabolism in sporulated oocysts during dormancy [41], [42]. So we thought even EtAMA1 transcripts were moderately expressed, EtAMA1 protein could remain a high expression in sporulated oocysts.

AMA1 has been an essential protein in apicomplexan invasion, which can be identified in invasive zoites [43]. qRT-PCR and Western Blotting analysis showed EtAMA1 was high expressed in sporozoites. Invasion inhibition assays revealed that rabbit antiserum against recombinant EtAMA1 blocked invasion of host cells by approximately 70%. Localization of EtAMA1 in DF-1 cells or in chicken ceca showed that the expression of EtAMA1 on the sporozoite surface increased when the parasites invaded the cells. These data therefore supported a more direct role for EtAMA1 in host invasion.

In the localization of EtAMA1, we observed the protein was not only found at the apical end, but also in the entire surface of the parasite even during invasion, which could also observed in *Toxoplasma* parasites [43]. Later during the parasite development in DF-1 cells, EtAMA1 was found on the merozoite surface and in the PV membrane. The PV is a crucial structure that protects the parasite against the antagonistic environment of the host cell [12]. When merozoites escape from mature schizonts to invade new host cells, they must pass through the PV membrane. Thus, we suggest that EtAMA1 has an important role in merozoite release.

In the sexual reproduction stage of *E. tenella*, EtAMA1 appeared to be expressed in gametocytes. However, not all the gametocytes examined expressed EtAMA1 and the protein was expressed in the form of numerous granules. In *E. tenella*, sexual reproduction involves a microgamete initiatively entering a macrogamete to form a zygote, which then develops into unsporulated oocyst. Therefore, the microgamete also requires an ‘invasion’ process. Many parasites, appearing to be zygotes in form, were observed in the ceca; therefore, we came to a conclusion that these gametocytes were microgametocytes with a high EtAMA1 expression, which needed further replication experiments. It may lead to the interesting observation that EtAMA1 is important not only in host invasion, but also in ‘self’ invasion.

In conclusion, we cloned and characterized the AMA1 of *E. tenella* and, as a result, have added significantly to current understanding of its role during parasite invasion. Given the importance of EtAMA1 in invasion and parasite development, this study is likely to have implications for both novel chemo- and immuno-therapeutic approaches to interfering with EtAMA1 function.

## References

[1] Shirley MW, Ivens A, Gruber A, Madeira AM, Wan KL (2004). The *Eimeria* genome projects: a sequence of events.. Trends Parasitol.

[2] Williams RB (1998). Epidemiological aspects of the use of live anticoccidial vaccines for chickens.. Int J Parasitol.

[3] Allen PC, Fetterer RH (2002). Recent advances in biology and immunobiology of *Eimeria* species and in diagnosis and control of infection with these coccidian parasites of poultry.. Clin Microbiol Rev.

[4] Williams RB (2006). Tracing the emergence of drug-resistance in coccidian (*Eimeria* spp.) of commercial broiler ﬂocks medicated with decoquinate for the first time in the United Kingdom.. Vet Parasitol.

[5] Morris GM, Woods WG, Richard DG, Gasser RB (2007). Investigating a persistent coccidiosis problem on a commercial broiler-breeder farm utilising PCR-coupled capillary electrophoresis.. Parasitol Res.

[6] Innes EA, Vermeulen AN (2006). Vaccination as a control strategy against the coccidial parasites *Eimeria, Toxoplasma* and *Neospora*.. Parasitology.

[7] Dalloul RA, Lillehoj HS (2006). Poultry coccidiosis: recent advancements in control measures and vaccine development.. Expert Rev Vaccines.

[8] Vermeulen AN, Schaap DC, Schetters TP (2001). Control of coccidiosis in chickens by vaccination.. Vet Parasitol.

[9] Shirley MW, Smith AL, Blake DP (2007). Challenges in the successful control of the avian coccidia.. Vaccine.

[10] Han HY, Lin JJ, Zhao QP, Dong H, Jiang LL (2010). Identification of differentially expressed genes in early stages of *Eimeria tenella* by suppression subtractive hybridization and cDNA microarray.. J Parasitol.

[11] Tabares E, Ferguson D, Clark J, Soon PE, Wan KL (2004). *Eimeria tenella* sporozoites and merozoites differentially express glycosylphosphatidylinositol-anchored variant surface proteins.. Mol Biochem Parasitol.

[12] Daszak P (1999). Zoite migration during *Eimeria tenella* infection: parasite adaptation to host defences.. Parasitol Today.

[13] Sasai K, Fetterer RH, Lillehoj H, Matusra S, Constantinoiu CC (2008). Characterization of monoclonal antibodies that recognize the *Eimeria tenella* microneme protein MIC2.. J Parasitol.

[14] Thomas AW, Deans JA, Mitchell GH, Alderson T, Cohen S (1984). The Fab fragments of monoclonal IgG to a merozoite surface antigen inhibit *Plasmodium knowlesi* invasion of erythrocytes.. Mol Biochem Parasitol.

[15] Alexander DL, Mital J, Ward GE, Bradley P, Boothroyd JC (2005). Identification of the moving junction complex of *Toxoplasma gondii*: a collaboration between distinct secretory organelles.. PLoS Pathog.

[16] Santos JM, Ferguson DJP, Blackman MJ, Soldati-Favre D (2011). Intramembrane cleavage of AMA1 triggers *Toxoplasma* to switch from an invasive to a replicative mode.. Science.

[17] Besteiro S, Michelin A, Poncet J, Dubremetz JF, Lebrun M (2009). Export of a *Toxoplasma gondii* rhoptry neck protein complex at the host cell membrane to form the moving junction during invasion.. PLoS Pathog.

[18] Lamarque M, Besteiro S, Papoin J, Roques M, Vulliez-Le Normand B (2011). The RON2-AMA1 interaction is a critical step in moving junction-dependent invasion by apicomplexan parasites.. PLos Pathog.

[19] Curtidor H, Patino LC, Arevalo-Pinzon G, Patarroyo ME, Patarroyo MA (2011). Identification of the *Plasmodium falciparum* rhoptry neck protein 5 (PfRON5).. Gene.

[20] Remarque EJ, Faber BW, Kocken CH, Thomas AW (2008). Apical membrane antigen 1: a malaria vaccine candidate in review.. Trends Parasitol.

[21] Donahue CG, Carruthers VB, Gilk SD, Ward GE (2000). The *Toxoplasma* homolog of *Plasmodium* apical membrane antigen-1 (AMA-1) is a microneme protein secreted in response to elevated intracellular calcium levels.. Mol Biochem Parasitol.

[22] Gaffar FR, Yatsuda AP, Franssen FFJ, Vries E (2004). Erythrocyte invasion by *Babesia bovis* merozoites is inhibited by polyclonal antisera directed against peptides derived from a homologue of *Plasmodium falciparum* apical membrane antigen 1.. Infect Immun.

[23] Zhang H, Compaore MK, Lee EG, Liao M, Zhang G (2007). Apical membrane antigen 1 is a cross-reactive antigen between *Neospora caninum* and *Toxoplasma gondii*, and the anti-NcAMA1 antibody inhibits host cell invasion by both parasites.. Mol Biochem Parasitol.

[24] Tonukari NJ (2010). *Theileria parva* apical membrane antigen-1 (AMA-1) shares conserved sequences with apicomplexan homologs.. Int J Biotech Mol Biol Res.

[25] Ng ST, Sanusi Jangi M, Shirley MW, Tomley FM, Wan KL (2002). Comparative EST analyses provide insights into gene expression in two asexual developmental stages of *Eimeria tenella*.. Exp Parasitol.

[26] Klotz C, Marhofer RJ, Selzer PM, Lucius R, Pogonka T (2005). *Eimeria tenella*: Identification of secretory and surface proteins from expressed sequence tags.. Exp Parasitol.

[27] Lal K, Bromley E, Oakes R, Prieto JH, Sanderson SJ (2009). Proteomic comparison of four *Eimeria tenella* life-cycle stages: unsporulated oocyst, sporulated oocyst, sporozoite and second-generation merozoite.. Proteomics.

[28] Blake DP, Billington KJ, Copestake SL, Oakes RD, Quail MA (2011). Genetic mapping identifies novel highly protective antigens for an apicomplexan parasite.. PLoS Pathog.

[29] Peroval M, Pery P, Labbe M (2006). The heat shock protein 90 of *Eimeria tenella* is essential for invasion of host cell and schizont growth.. Int J Parasitol.

[30] Tomley F (1997). Techniques for isolation and characterization of apical organelles from *Eimeria tenella* sporozoites.. Methods.

[31] Shirley MW (1995). *Eimeria* species and strains of chickens. In Biotechnology Guidelines on Techniques in Coccidiosis Research.. The European Commission DGXII, Luxembourg City, Luxembourg, 24.

[32] Xie MQ, Gilbert JM, Fuller AL, McDougald LR (1990). A new method for purification of *Eimeria tenella* merozoites.. Parasitol Res.

[33] Jiang LL, Lin JJ, Han HY, Dong H, Zhao QP (2011). Identification and partial characterization of a serine protease inhibitor (serpin) of *Eimeria tenella*.. Parasitol Res.

[34] Kimbitaa EN, Xuan X, Huang X, Miyazawa T, Fukumoto S (2001). Serodiagnosis of *Toxoplasma gondii* infection in cats by enzyme-linked immunosorbent assay using recombinant SAG1.. Vet Parasitol.

[35] Jiang LL, Lin JJ, Han HY, Dong H, Zhao QP (2011). Establishment and application of DF-1 cell culture for the sporozoites of *Eimeria tenella*.. Chin Vet Sci.

[36] Jahn D, Matros A, Bakulina AY, Tiedemann J, Schubert U (2009). Model structure of the immunodominant surface antigen of *Eimeria tenella* identified as a target for sporozoite-neutralizing monoclonal antibody.. Parasitol Res.

[37] Li L, Brunk BP, Kissinger JC, Pape D, Tang K (2003). Gene discovery in the apicomplexa as revealed by EST sequencing and assembly of a comparative gene database.. Genome Res.

[38] Pizarro JC, Vulliez-Le Normand B, Chesne-Seck ML, Collins CR, Withers-Martinez C (2005). Crystal structure of the malaria vaccine candidate apical membrane antigen 1.. Science.

[39] Hodder AN, Crewther PE, Anders RF (2001). Specificity of the protective antibody response to apical membrane antigen 1.. Infect Immun.

[40] Dutta S, Lalitha PV, Ware LA, Barbosa A, Moch JK (2002). Purification, characterization, and immunogenicity of the refolded ectodomain of the *Plasmodium falciparum* apical membrane antigen 1 expressed in *Escherichia coli*.. Infect Immun.

[41] Belli SI, Walker RA, Flowers SA (2005). Global protein expression analysis in apicomplexan parasites: Current status.. Proteomics.

[42] Wilson PAG, Fairbairnz D (1961). Biochemistry of sporulation in oocysts of *Eimeria acervulina*.. J Eukaryot Microbiol.

[43] Tyler JS, Treeck M, Boothroyd JC (2011). Focus on the ringleader: the role of AMA1 in apicomplexan invasion and replication.. Trends Parasitol.

